# Two new species of *Striglina* Guenée, 1877 (Lepidoptera, Thyrididae) from China

**DOI:** 10.3897/BDJ.12.e126268

**Published:** 2024-05-21

**Authors:** Feiran Chen, Yulong Zhang, Min Wang

**Affiliations:** 1 South China Agricultural University, Guangzhou, China South China Agricultural University Guangzhou China

**Keywords:** morphology, Nanling, Striglininae, Yunnan

## Abstract

**Background:**

The genus *Striglina* is the most species-rich genus in the subfamily Striglininae, which includes about 80 species and subspecies and it has always been a popular research taxon within Thyrididae.

**New information:**

Two new species of the genus *Striglina* Guenée, 1877, *S.whalleyi* **sp. nov.** and *S.pararubricans* **sp. nov.** from China are described and illustrated. *Striglinawhalleyi* **sp. nov.** is similar to *S.irresectaobscura* Whalley, 1976, but its wing ground colour is lighter and the sacculus process is shorter. *Striglinapararubricans* **sp. nov.** is similar to *S.rubricans*, but the fore-wing is narrower, the uncus processes and sacculus process are longer. Holotypes are deposited in the Department of Entomology, College of Plant Protection, South China Agricultural University, Guangzhou.

## Introduction

The genus *Striglina* was established by Guenée in 1877 with *S.lineola* Guenée, 1877 as its type species. To avoid replacing a better-known generic name by another unused name, the International Commission on Zoological Nomenclature placed the name *Striglina* on the Offical List instead of the order generic name *Daristane* Walker, 1850 (Nye 1974). Whalley (1976) revised the genus *Striglina* and defined its generic characters as follows: eyes without interfacetal hairs; antennae usually minutely ciliate; proboscis present; labial palps 3-segmented; fore tibia with epiphysis; hind tibia with two pairs of spines; tarsi with spines, usually rows, sometimes apical groups of three; hind-wing with Sc+R_1_ and Rs free, occasionally joined for part of the length; uncus modified; gnathos with peg-like teeth; valva reduced; subscaphium with long scales or strongly sclerotised; female with triple frenula, rarely double; ostial plate highly sclerotised; bursa duct often long, convolute; spiny signum usually present in bursa. Chu and Wang (1991) studied 16 species of *Striglina* from China including 14 new species and subspecies. Afterwards, Inoue (1998) synonymised *S.bispota* Chu & Wang, 1991 with *S.propatula* Whalley, 1974 and *S.elaphra* Chu & Wang, 1991 with *S.mediofascia* Swinhoe, 1906. In the book "Moths of Guangdong Nanling National Nature Reserve", Owada and Wang (2011) found two new species of *Striglina* and synonymised *S.hala* Chu & Wang, 1991 with *S.irresecta* Whalley, 1976 and *S.stricta* Chu & Wang, 1991 with *Sonagarastrigipennis* Moore, 1882. Later, Owada et al. (2016) described four new species of *Striglina* from China and North Vietnam; they synonymised S. suzukii szechwanensis with *S.duplicifimbria* and raised *S.duplicifimbriacerta* to species level. Subsequently, Owada (2017) and Owada (2019) revised the *Striglinacancellata* complex in the *S.venia* species-group and reviewed fourteen species of the subfamily Striglininae from Taiwan. Most recently, Owada (2023) studied the *tibiaria*-group of *Striglina* and discussed the origin and evolution of the male scent organs and ovoviviparity. Before this paper, the genus included about 80 species and subspecies around world (Whalley 1976, Owada et al. 2016, Owada 2017, Owada 2019, Owada 2023). In this paper, we added two new species to Chinese fauna.

## Materials and methods

The specimens were collected using a light trap. Adults were photographed by a NIKON CoolPix S7000 digital camera. Abdomens were removed and macerated in hot 10% sodium hydroxide (NaOH) solution for examination of genitalia, photographs of which were taken under a Zeiss SteReo Discovery V.12. Adults and genitalia photos were all processed by Adobe Photoshop CC2018 software. Terminology of adult and genitalia follows Whalley (1976).

## Taxon treatments

### Striglina whalleyi sp. nov.

1EB6334C-15F1-5794-97FB-FD9BC44C0668

106FD8E0-0B2D-4D6A-82FD-01789F81D5A7

#### Materials

- **Type status:** Holotype. **Occurrence:** recordedBy: Hai-Ming Xu; sex: male; occurrenceID: 65C1AE97-57B7-50AD-8DEA-5D4DC03F3E10; **Location:** country: China; stateProvince: Yunnan; county: Chuxiong; locality: Yipinglang; **Event:** eventDate: 10 Apr 2013
- **Type status:** Paratype. **Occurrence:** recordedBy: Hai-Ming Xu; sex: 2 males; occurrenceID: 34006CB0-52AE-5EA6-A1AD-BCAD2408B30E; **Location:** country: China; stateProvince: Guangdong; county: Shaoguan; locality: Nanling; **Event:** eventDate: 05 Jun 2011

#### Description

Male (Fig. 1a). Wingspan 23-25 mm. Antennae filiform, brown; head dark brown; thorax, tegulae and legs brownish-yellow; abdomen dorsally brown. Fore-wing ground colour brownish-yellow with dark brown posterior line from near apex to middle of inner margin, near the discal cell with two large dark puncta; cilia whitish-yellow, terminally black. Hind-wing ground colour brownish-yellow, median line indistinct at costa.

Male genitalia (Fig. 3a). Uncus bifid apically, with two shorter processes; two lateral processes apically curved inwards. Subscaphium hairy. Gnathos strip-type with serried tooth. The valvae weak membranous straps; median valval process weak. Sacculus process short sclerotised, enlarged apically. Aedeagus short and broad with a cluster of cornuti.

Female: unknown.

#### Diagnosis

This new species resembles *S.irresectaobscura* Whalley, 1976, but the ground colour of wings is yellow (reddish-brown in the latter, *Fig. 1a, b*), the oblique line from apex to middle of dorsum is straight, dark brown (ochre in the latter). In male genitalia, the uncus is broader, the sacculus process is shorter than *S.irresectaobscura*, the aedeagus is broader basal (Fig. 3a, b).

#### Etymology

The species is dedicated to Mr. Paul Ernest Sutton Whalley, in honour of his marvellous work on Thyrididae.

#### Distribution

China: Yunnan (Chuxiong), Guangdong (Shaoguan).

#### Notes

Our study shows that *S.irresectaobscura* seems more closely related to the new species than to *S.irresectairresecta* morphologically and zoogeographically. Judging from the holotype pictures (Fig. 2) from the Natural History Museum (2014), the hind-wing pattern of *S.irresecta* is also showing the same characteristics.

### Striglina pararubricans sp. nov.

CDF45F60-0715-5D97-800C-96395DF4066D

6727C682-0907-412B-81C3-AF04A368293F

#### Materials

- **Type status:** Holotype. **Occurrence:** recordedBy: Hai-Ming Xu & Min Wang; sex: male; occurrenceID: 9961BA4D-9369-5A38-B7A3-9264FBAA4506; **Location:** country: China; stateProvince: Guangdong; county: Shanguan; locality: Nanling; verbatimElevation: 1249 m; **Event:** eventDate: 09 Apr 2011
- **Type status:** Paratype. **Occurrence:** recordedBy: Min Wang; sex: male; occurrenceID: BE9D53B6-749E-5F55-AE6D-90B239570504; **Location:** country: China; stateProvince: Guangdong; county: Shanguan; locality: Nanling; **Event:** eventDate: 17 Feb 2006
- **Type status:** Paratype. **Occurrence:** recordedBy: Hai-Ling Zhuang; sex: male; occurrenceID: AF2BE3D0-8F4F-5BC7-BFBD-CBC29B9E7605; **Location:** country: China; stateProvince: Guangdong; county: Shanguan; locality: Nanling; **Event:** eventDate: 27 Mar 2012

#### Description

*Male* (Fig. 1c). Wingspan 28-30 mm. Antennae filiform, dark brown; head ochrous, labial palpus long, upcurved, ochrous brown; thorax and tegulae covered in ochrous scales; abdomen dorsally dark brown. Fore-wing ground colour whitish ochrous with a broad dark brown striation from apex to middle of hind margin, reticulate pattern prominent. Hind-wing ground colour light brown, wing pattern not as obvious as fore-wing.

*Male genitalia* (Fig. 3c). Uncus slightly bifid apically, longer with two side processes. Subscaphium hairy. Gnathos strip-type with serried tooth. The valvae weak membranous elongated strips, median valval process hook-shaped, strongly sclerotised. Sacculus process sclerotised, apical part bifid, basal part moderately broader than apical part. Aedeagus short and broad with two clusters of cornuti.

*Female*: unknown.

#### Diagnosis

This wing pattern of the new species resembles *S.rubricans* Owada et Huang, 2016, but the fore-wing is narrower and the outer margin is curved (triangular fore-wing in *rubricans Fig. 1c, d*), posterior line with a large dark brown area, the uncus is thinner and two processes are longer, the median valval process is thinner and more sclerotised, the sacculus process is slender (Fig. 3c, d).

#### Etymology

The specific name *pararubricans* is derived from the other specific name *rubricans* due to the resemblance of the present new species in morphological characters to the species *S.rubricans*.

#### Distribution

China: Guangdong (Shaoguan).

## Supplementary Material

XML Treatment for Striglina whalleyi

XML Treatment for Striglina pararubricans

## Figures and Tables

**Figure 1a.**
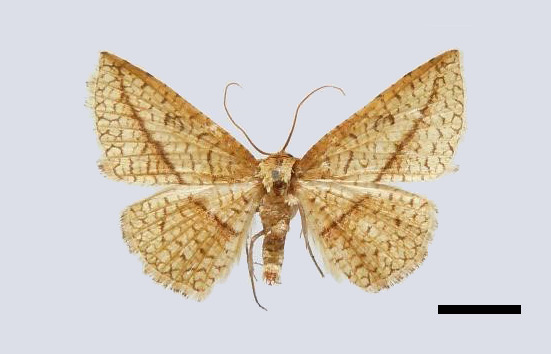
*S.whalleyi* sp. nov., holotype male, Yunnan;

**Figure 1b.**
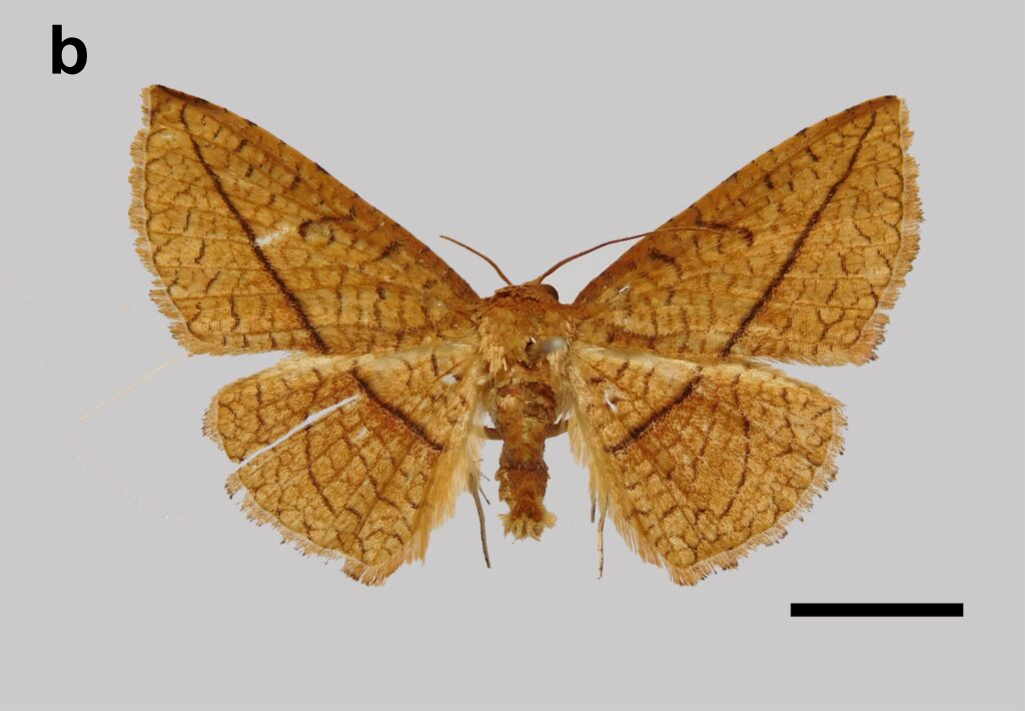
*S.irresectaobscura* Whalley, 1976, male, Xizang;

**Figure 1c.**
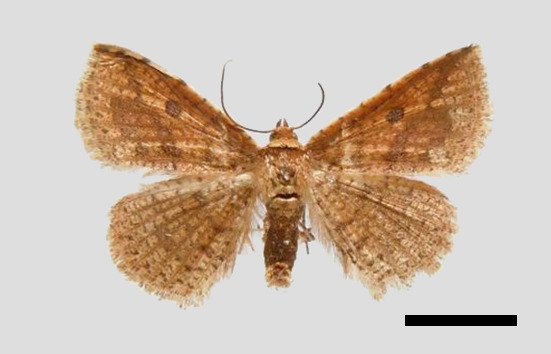
*S.pararubricans* sp. nov., holotype male, Guangdong;

**Figure 1d.**
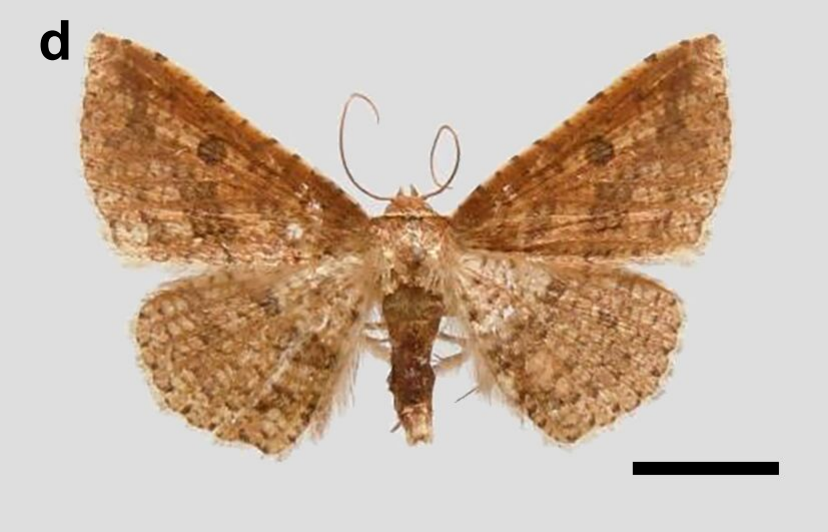
*S.rubricans* Owada et Huang, 2016, paratype male, Guangdong.

**Figure 2a.**
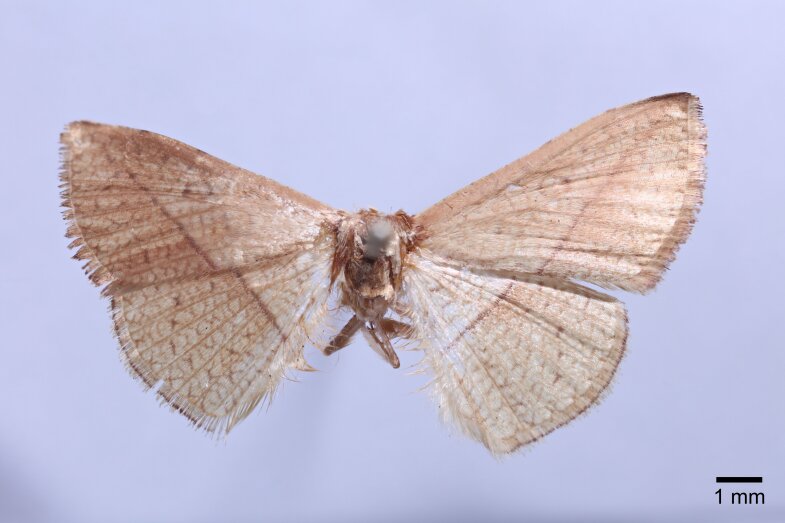
*S.irresectairresecta* Whalley, 1976, male, holotype, East Pegu, India, adult dosal view (NHMUK013698289, ©The trustees of NHMUK);

**Figure 2b.**
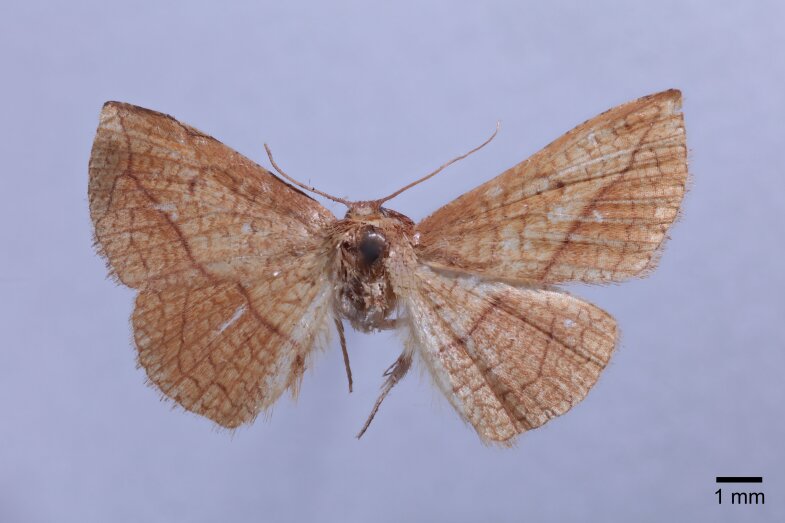
*S.irresectaobscura* Whalley, 1976, male, holotype, Pedong (*Desgodins*), India, adult dorsal view (NHMUK013698290, ©The trustees of NHMUK);

**Figure 2c.**
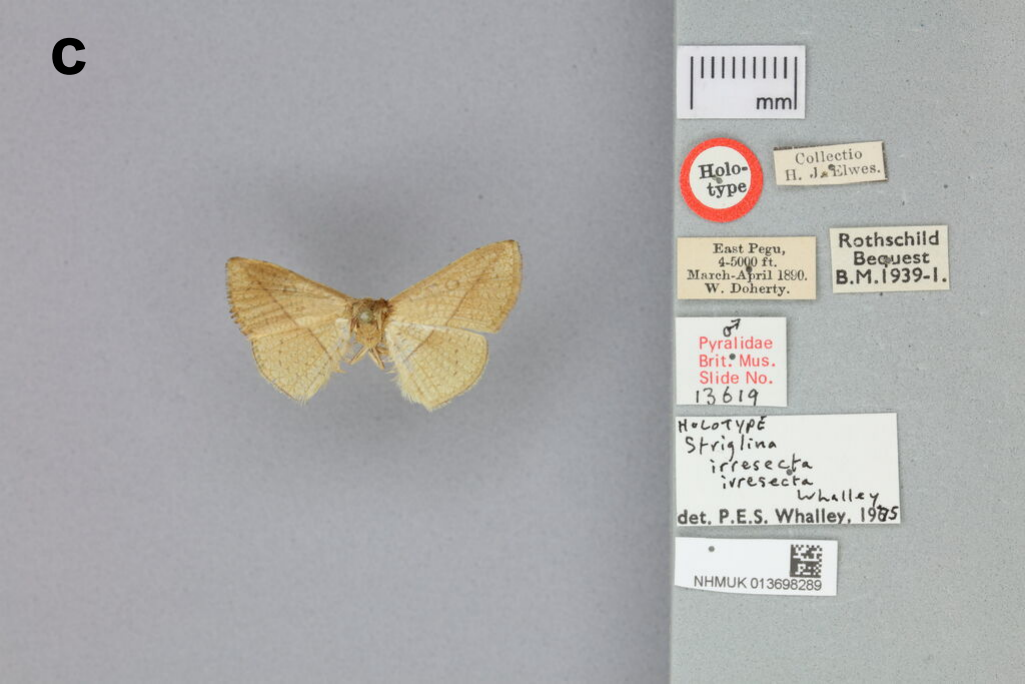
*S.irresectairresecta* Whalley, 1976, holotype with label (NHMUK013698289, ©The trustees of NHMUK);

**Figure 2d.**
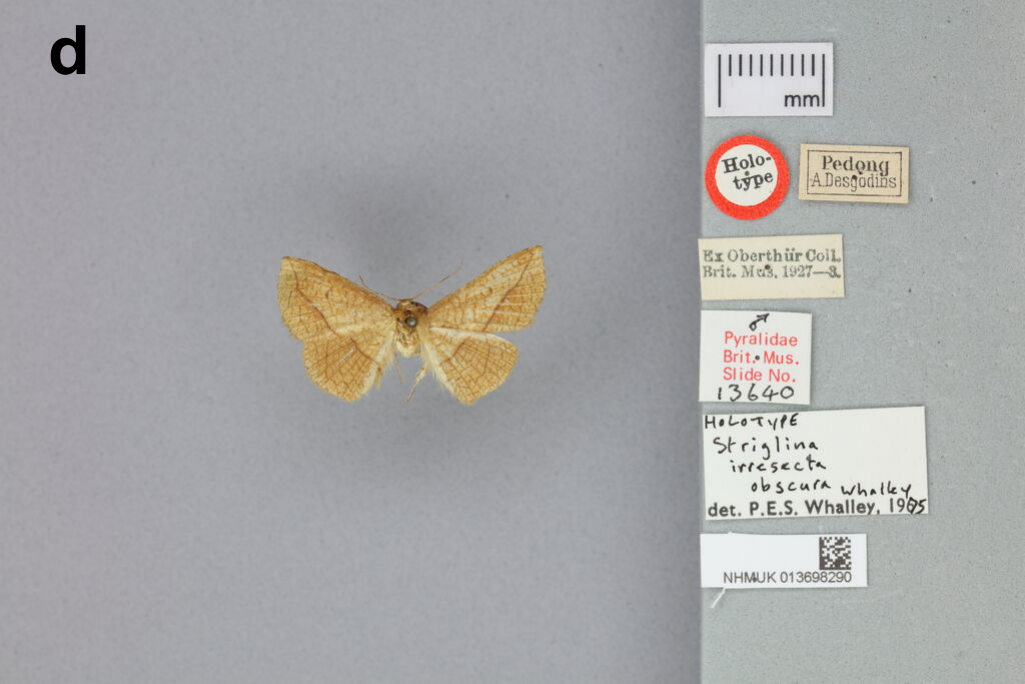
*S.irresectaobscura* Whalley, 1976, holotype with label (NHMUK013698290, ©The trustees of NHMUK).

**Figure 3a.**
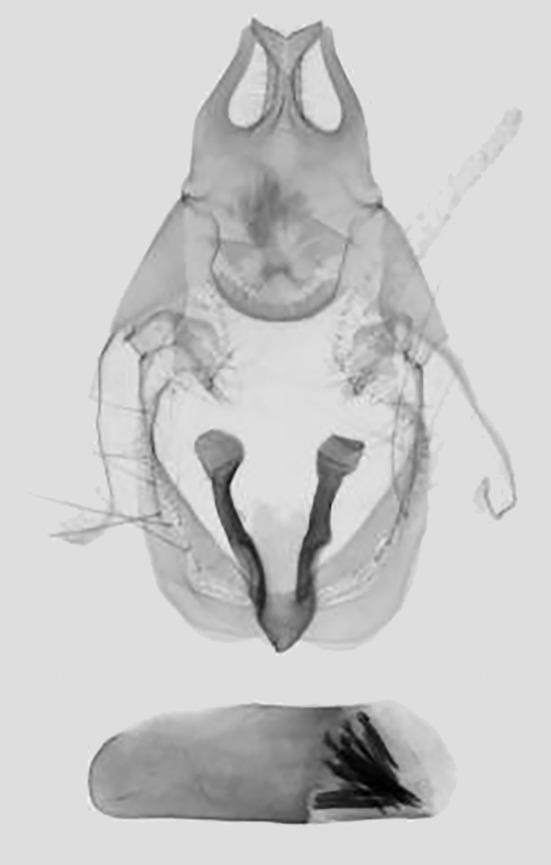
*S.whalleyi* sp. nov., holotype male, Yunnan;

**Figure 3b.**
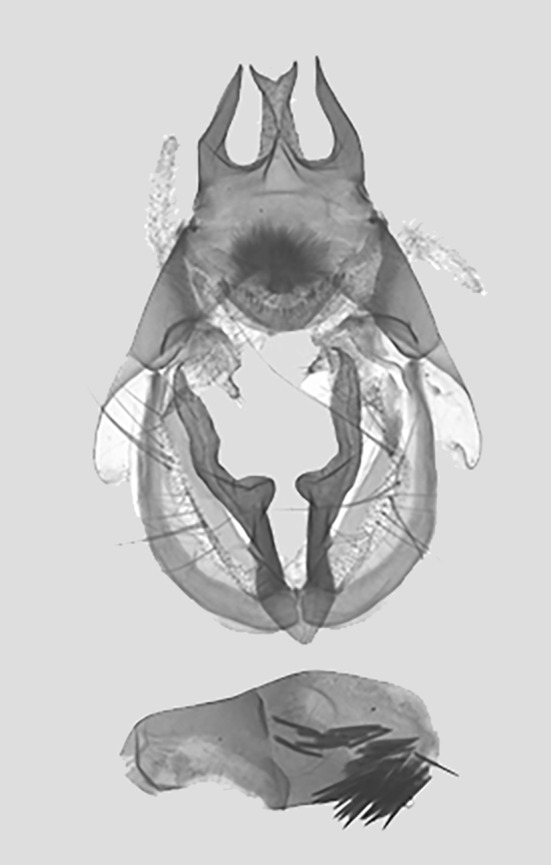
*S.irresectaobscura* Whalley, 1976,, Xizang;

**Figure 3c.**
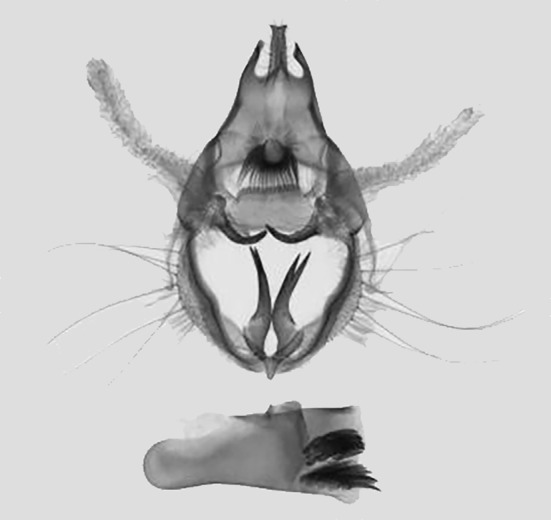
*S.pararubricans* sp. nov., holotype, Guangdong;

**Figure 3d.**
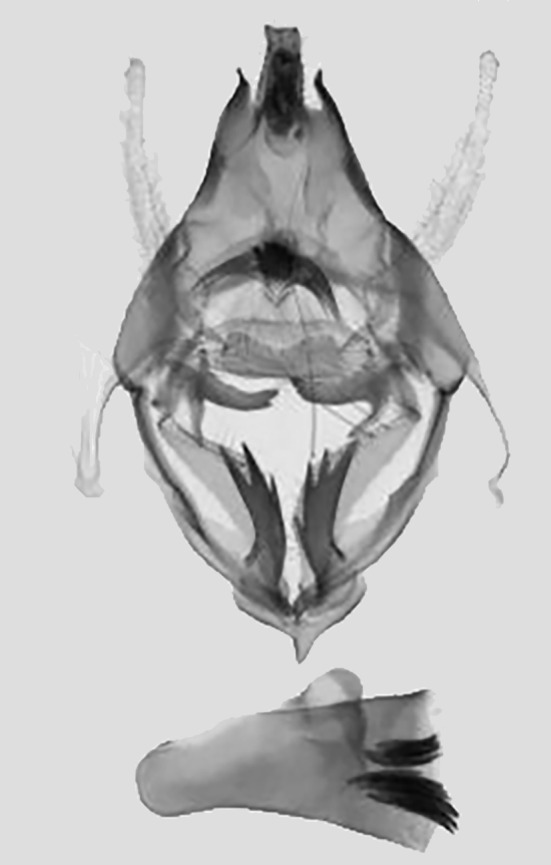
*S.rubricans* Owada et Huang, 2016, paratype, Guangdong.
